# Trends in pregabalin misuse and abuse: A 25-year bibliometric perspective

**DOI:** 10.5937/jomb0-59752

**Published:** 2026-01-06

**Authors:** Saliha Aksun, Karalar Furkan Oguz, Mert Üge, Berna Cafer

**Affiliations:** 1 Izmir Katip Celebi University, Medical Faculty, Department of Medical Biochemistry, Atatürk Education and Research Hospital, Department of Clinical Biochemistry, Izmir, Türkiye; 2 Department of Clinical Biochemistry, Karabuk Public Health Laboratory, Karabuk, Türkiye; 3 Ege University Health Sciences Institute, Izmir, Türkiye

**Keywords:** pregabalin, gabapentinoids, misuse, abuse, addiction, pregabalin, gabapentinoidi, neadekvatna upotreba, zloupotreba, zavisnost

## Abstract

**Background:**

Originally classified among antiepileptic drugs, pregabalin has been prescribed for clinical conditions such as neuropathic pain, generalised anxiety disorder, and fibromyalgia. In recent years, accumulating evidence has highlighted its potential for misuse and abuse, particularly among individuals with a high prevalence of opioid dependence. This study aims to conduct a comprehensive bibliometric analysis of global research on pregabalin misuse and abuse, to identify key trends, influential contributors, and collaborative networks.

**Methods:**

Bibliometric design was employed using data retrieved from the Web of Science Core Collection database. The search was conducted in February 2025, and relevant publications were identified based on predefined inclusion and exclusion criteria. A total of 449 eligible records were exported and compiled for analysis. The bibliometric analysis was carried out using the bibliometrix R package and its Web-based interface Biblioshiny, both operating within the RStudio environment.

**Results:**

The analysis revealed a notable increase in scientific output on pregabalin misuse and dependence, particularly after 2010. The United States emerged as the most prolific and central country within the global collaboration network, followed by the United Kingdom, Germany, and France. Leading authors and institutions were concentrated in specific academic clusters. Keywords such as "abuse," "addiction," and "opioids" have shown an increasing trend since 2016.

**Conclusions:**

This study highlights the growing academic interest in the misuse and abuse potential of pregabalin, particularly over the past decade. The findings reveal a centralised research structure, with significant contributions from high-capacity countries such as the United States, the United Kingdom, and Germany. Emerging participation from developing countries, including Türkiye, underscores a broadening global awareness of the issue. The results emphasise the importance of interdisciplinary collaboration, policies, and the critical role of clinical laboratories in monitoring pregabalin-related risks.

## Abstract (sr)

**Uvod:**

Pregabalin, prvobitno klasifikovan kao antiepileptik, se prepisuje za klinička stanja kao sto su neuropatski bol, generalizovani anksiozni poremećaj i fibromialgija. Tokom poslednjih godina, sve vise dokaza ukazuje na njegov potencijal za neadekvatnu upotrebu i zloupotrebu, naročito među osobama sa visokom prevalencijom zavisnosti od opioida. Cilj ove studije je sprovođenje sveobuhvatne bibliometrijske analize globalnih istraživanja o zloupotrebi i neadekvatnoj upotrebi pregabalina, sa fokusom na identifikaciju ključnih trendova, uticajnih autora i mreža saradnje.

**Metode:**

Bibliometrijski dizajn je primenjen korišćenjem podataka preuzetih iz baze Web of Science Core Collection. Pretraga je obavljena u februaru 2025. godine, a relevantne publikacije su identifikovane na osnovu unapred definisanih kriterijuma za uključivanje i isključivanje. Ukupno je 449 relevantnih radova izvezeno i obrađeno za analizu. Bibliometrijska analiza je sprovedena pomoću paketa "bibliometrix" u R okruženju i njegovog veb interfejsa "Biblioshiny", unutar RStudio platforme.

**Rezultati:**

Analiza je pokazala značajan porast naučnih radova o zloupotrebi i zavisnosti od pregabalina, posebno nakon 2010. godine. Sjedinjene Američke Države su se izdvojile kao najproduktivnija i centralna zemlja u globalnoj mreži saradnje, a zatim slede Ujedinjeno Kraljevstvo, Nemačka i Francuska. Vodeći autori i institucije koncentisani su u određenim akademskim klasterima. Ključne reči poput "zloupotreba", "zavisnost" i "opioidi" beleže porast od 2016. godine.

**Zaključak:**

Ova studija ističe rastuće akademsko interesovanje za potencijal zloupotrebe pregabalina, posebno u poslednjoj deceniji. Rezultati otkrivaju centralizovanu strukturu istraživanja, sa najvećim doprinosima iz zemalja sa visokim istraživačkim kapacitetima kao što su SAD, Ujedinjeno Kraljevstvo i Nemačka. Pojava učešća zemalja u razvoju, uključujući Tursku, ukazuje na širenje globalne svesti o ovom problemu. Rezultati naglašavaju značaj interdisciplinarne saradnje, politika i ključnu ulogu kliničkih laboratorija u praćenju rizika povezanih sa pregabalinom.

## Introduction

Pregabalin is a second-generation antiepileptic drug that has been widely used in the treatment of epilepsy, diabetic neuropathy, fibromyalgia, and generalised anxiety disorder. In recent years, increasing concerns have emerged regarding its non-medical use and the ease of obtaining it without a prescription [1]. Although the addictive potential of gabapentinoids (pregabalin and gabapentin) at therapeutic doses is generally considered low, high doses of pregabalin have been reported to induce euphoria, increase energy and relaxation, alleviate opioid withdrawal symptoms, and potentiate the effects of other substances. These effects may contribute to the misuse of pregabalin, particularly among individuals with a histor y of opioid dependence [2] [3].

Notably, while the prevalence of gabapentinoid misuse in the general population is approximately 1%, the reported rate of pregabalin misuse among individuals with opioid use disorder ranges from 3% to 68% [3]. In light of these alarming trends, several countries, most notably the United Kingdom (UK), have implemented regulatory measures to control pregabalin and gabapentin; for instance, in 2019, gabapentinoids were reclassified as Class C controlled substances in the UK [4]. As regulatory authorities have tightened control over pregabalin, academic research into its misuse has expanded considerably [2] [5].

In this context, bibliometric analysis is a powerful method that enables the quantitative evaluation of scientific publications on a specific topic. By revealing trends in the literature, the most productive authors, countries, institutions, highly cited studies, and academic collaborations, bibliometric analysis helps to understand the current scientific landscape and provides strategic guidance for future research. Moreover, it contributes to the mapping of interdisciplinary dynamics and the evaluation of scientific impact [6].

This study presents a bibliometric analysis of the literature on pregabalin misuse, based on publications containing the keywords »pregabalin misuse«, »pregabalin abuse«, »pregabalin addiction«, and »gabapentinoid misuse«. The objective is to identify prevailing research trends, influential studies, and major collaborations in the field, thereby guiding future investigations.

## Materials and methods

### Research strategy

In this study, the Web of Science Core Collection (WoSCC) was utilised as the bibliometric data source due to its compatibility with the programs used, its provision of comprehensive bibliographic data, its support for citation relationship analysis, and its recognition as one of the most widely accepted scientific databases in the field. The data cover the literature published between 2001 and 2025. A total of 662 records were retrieved during the initial search. The inclusion criteria for this study were as follows: publications published between 2001 and 2025, written in the English language, categorised as either »article« or »review article« in terms of article type, and containing at least one of the predefined keywords. The exclusion criteria included book chapters, conference papers, case reports, commentaries, short communications, letters to the editor, and publications written in languages other than English. Based on these inclusion and exclusion criteria, a total of 449 publications met the eligibility requirements and were included in the bibliometric analysis.

### Data analysis

All bibliographic data obtained from the WoSCC in February 2025 were exported in batches of 500 records and subsequently merged into a single file before analysis. The collected data were analysed using the open-source Bibliometrix R package (Italy, University of Naples Federico II) and its web-based graphical interface, Biblioshiny, both of which run in the R programming environment, typically accessed through RStudio (Posit Software, USA). Bibliometrix is a comprehensive R package that enables the systematic analysis of the bibliometric characteristics of scientific publications [7]. In addition to code-based analyses, the analysis process was also conducted through the Biblioshiny interface. The .bib file obtained from the Web of Science database was uploaded into the interface, after which fundamental bibliometric indicators related to publication count, citation frequency, authors, and sources were compiled. The annual distribution of publications, the most influential journals and authors were evaluated, and keyword-based word clouds and trend graphs were generated. International collaboration maps were visualised through co-authorship networks, and keyword clusters and thematic development processes were examined using thematic maps. All graphical outputs were exported in .jpg format, and the resulting data were interpreted to visualise literature trends, research gaps, and patterns of international collaboration. In this context, structures that play a bridging role in the flow of information were identified, particularly using the betweenness centrality metric. Nodes with a betweenness centrality value greater than 0.1 were considered »key actors« occupying strategic positions within the network [8]. Clustering quality was assessed using the Q-modularity value, where a range between 0.4 and 0.8 indicates a good level of thematic clustering. Cluster homogeneity was measured using the mean silhouette value, with values greater than 0.7 indicating clusters with high internal consistency [9].

## Results

### Annual publication trends

**Figure 1:**
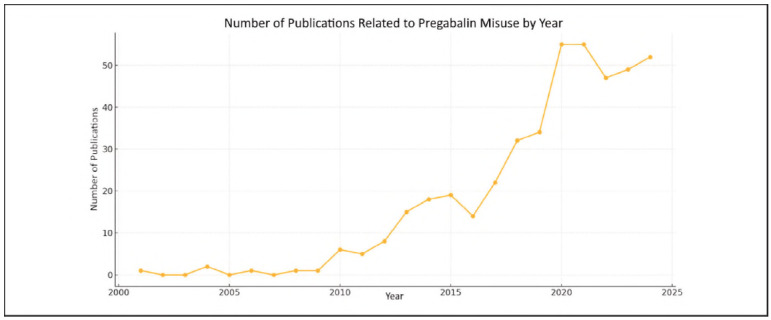
Annual distribution of publications on pregabalin misuse.

An analysis of the annual distribution of academic publications on pregabalin misuse and dependence reveals a clear upward trend in the literature. While the number of studies on this topic was negligible in the early 2000s, a gradual increase in publications has been observed since 2010 [10] [11]. As shown in Figure 1, a steady increase in the number of publications has been observed since 2014. The period between 2018 and 2021 represents the peak in publication activity on this topic, with an average of approximately 50 publications per year, reaching a maximum of 55 publications in both 2020 and 2021. Overall, the publication trend indicates that the topic of pregabalin misuse has gained increasing importance over the past decade, with a continuous growth in research volume and an annual growth rate of 10.91%.

### Top contributing authors and institutions

**Figure 2:**
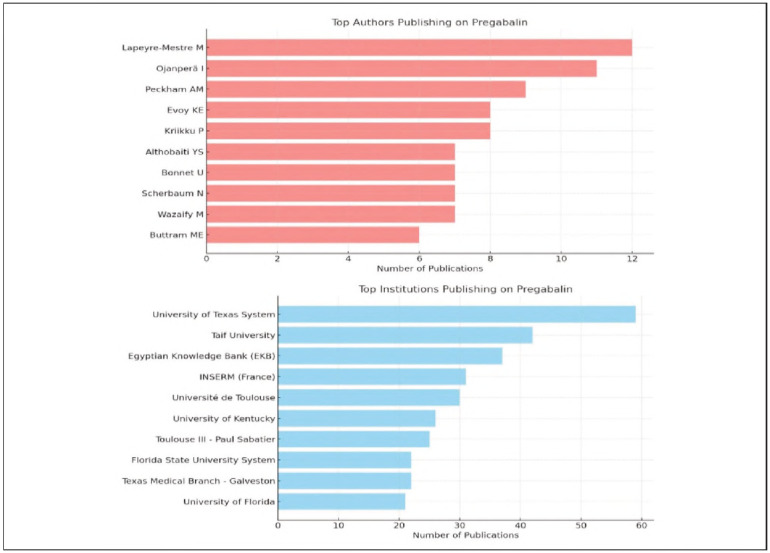
Top authors and institutions publishing on pregabalin misuse.

An analysis of the authors contributing to the literature on pregabalin misuse revealed that key studies in the field are concentrated around a limited number of researchers. As shown in Figure 2, Lapeyre-Mestre M ranks first with 12 publications, followed by Ojanpera I with 11 publications and Peckham AM with 9 publications.

From an institutional perspective, the University of Texas System, as shown in Figure 2, stands out as the most productive institution by far, with a total of 59 publications. Taif University (Saudi Arabia) ranks second with 42 publications and is particularly notable for its recent focus on substance misuse and psychiatric disorders in the Middle East region [5].

### Top contributing countries

The United States (US) is by far the leading country in terms of scientific publications on pregabalin misuse, dependence, and related topics. With a total of 128 publications, the US accounts for a substantial portion of the literature. The UK follows with 43 publications, and Germany ranks third with 26. These two European countries have produced significant research on the safety and misuse of gabapentinoids, likely due to their strong pharmacovigilance systems and well-established multidisciplinary research infrastructures [10]. France (25 publications), Australia (21), and Italy (17) also stand out as key contributors. Türkiye ranks among the top 10 countries, with a total of 16 publications related to pregabalin.

### Sources and citation impact in pregabalin research: a bibliometric analysis

The British Journal of Clinical Pharmacology is the most prolific journal in this field, publishing 11 articles on the topic. It is followed by Drug and Alcohol Dependence, Frontiers in Psychiatry, and the Journal of Analytical Toxicology, each with 10 publications, positioning them as key platforms in the literature. The studies published in these journals predominantly address the effects of pregabalin within the context of substance dependence and its toxicological outcomes. Schifano F [1] is the most cited author with a total of 135 citations. He is followed by Ojanpera I with 111 citations and Lapeyre-Mestre M with 109 citations. Peckham AM (101 citations) and Evoy KE (82 citations) have contributed significantly through systematic reviews, particularly in studies originating from the US, by examining the misuse profiles of gabapentinoids in conjunction with opioids [2] [3].

At the top of the list is Schifano F's [1] article, »*Misuse and abuse of pregabalin and gabapentin: cause for concern?*« (2014, CNS Drugs), which is the most cited study in the field with 72 citations [11]. The second most cited is Schwan S. [12] (2010, European Journal of Clinical Pharmacology), followed by Grosshans M. [13] (2013, European Journal of Clinical Pharmacology). These studies have contributed significantly to the literature by highlighting the clinical use characteristics, psychoactive effects, and potential addictive properties of pregabalin [12] [13]. The study by Hakkinen M. [14] (2014, Forensic Science International), which investigated the toxicological role of pregabalin in fatal cases in Finland, has made a significant contribution to the field with 67 citations. Similarly, the publications by Chiappini S. (2016, CNS Drugs) and Lyndon A. (2017, Addiction) are also frequently cited, as they document the rising trend in gabapentinoid misuse [10] [15].

### Keyword analysis and trends

The most frequently occurring keyword is »pregabalin« (175 occurrences), which directly reflects the central focus of the study. It is followed by »gabapentin« (83 occurrences), indicating that the gabapentinoid group in general has been a prominent subject of research.

The terms »gabapentinoids« and »gabapentinoid« suggest that the entire class of molecules has been systematically investigated. In addition, keywords such as »neuropathic pain« (18), »chronic pain« [12], and »pain« [15] indicate that pregabalin is frequently addressed in the context of its clinical uses, particularly in the management of neuropathic and chronic pain.

**Figure 3:**
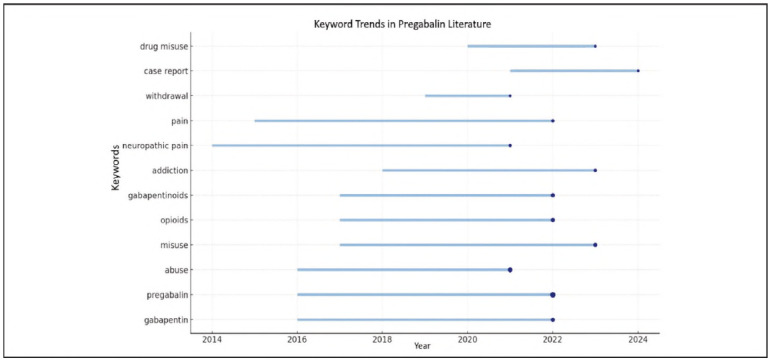
Temporal distribution of the most frequently used keywords in publications.

As shown in Figure 3, an examination of keyword trends reveals that terms such as »gabapentin,« »pregabalin,« »abuse,« »misuse,« and »opioids« have been increasingly used since 2016. These concepts have formed the foundation of studies addressing both the pharmacological properties of the drug and its potential for misuse [3].

### Collaboration networks among countries and authors

**Figure 4:**
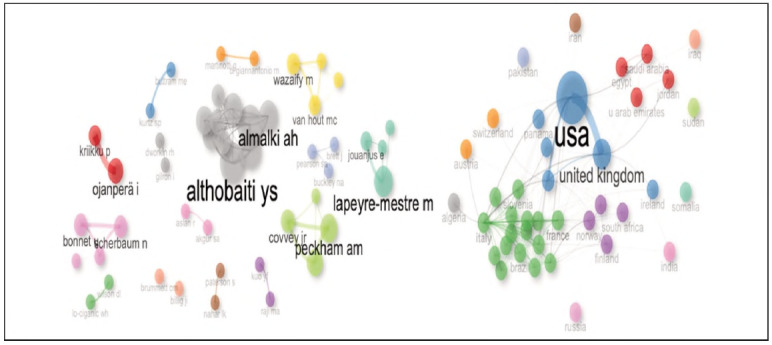
Collaboration network map of authors and countries.

The collaborative structures within the pregabalin-related literature were evaluated using bibliometric analysis, focusing on the relationships among countries, authors, and institutions. As shown in Figure 4, these relationships were examined based on centrality metrics, which serve as key indicators of an actor's strategic position within the network and the strength of its connections to other nodes.

According to the analysis, the US exhibited the highest centrality value (0.45), indicating its dominant position within the international research network and its leading role in collaborative efforts. As shown in Table 1, in 2021, Evoy KE from the US emerged as a leading author with five publications, while the University of Texas System stood out as the most productive institution with a total of 59 publications.

**Table 1:** Collaboration patterns between countries, authors, and institutions.

Country	Centrality	Author	n	Institution	n
USA	0.45	Evoy KE	5	University of Texas System (USA)	59
France	0.38	Lapeyre-Mestre M	6	Taif University (Saudi Arabia)	30
UK	0.35	Schifano F	4	Egyptian Knowledge Bank (EKB)	21
Australia	0.25	Cairns R	3	INSERM (France)	18
Turkey	0.19	Alan BE	2	Université de Toulouse (France)	15
Germany	0.15	Grosshans M	3	University of Kentucky (USA)	14
Finland	0.12	Hakkinen M	2	Toulouse III - Paul Sabatier (France)	13
Saudi Arabia	0.1	Alkhalaf AA	2	Florida State University System (USA)	12

France and the UK followed the US with centrality values of 0.38 and 0.35, respectively. Lapeyre-Mestre M from France and Schifano F from the UK are among the leading authors in the field.

Among the countries with moderate centrality values, Australia (0.25) and Türkiye (0.19) stand out. Cairns R (Australia) and Alan BE (Türkiye) have been identified as key contributors to the literature from their respective countries.

## Discussion

Based on a bibliometric analysis of the literature evaluating the misuse and addiction potential of pregabalin, this study offers important insights from both scientific and clinical perspectives. The findings indicate that, in recent years, there has been a significant increase in awareness within the literature regarding the rising rates of pregabalin misuse, rather than its therapeutic effects. Notably, a marked increase in publications focusing on this topic has been observed since 2010, and this growth has extended beyond case reports to include multidisciplinary and international collaborations.

The US serves as the central hub of the global research network, both in terms of publication volume and centrality value. Systematic reviews by authors such as Evoy KE and Peckham AM on the misuse profiles of gabapentinoids carry significant weight in the literature and play a key role in shaping the direction of research in this field. In particular, Evoy's 2021 study has comprehensively documented the growing trend of pregabalin misuse among opioid users. This work provides a valuable source of data not only for addiction medicine but also for informing public health policy [2] [3].

France-based studies, led primarily by Lapeyre-Mestre M, have been shaped particularly through the analysis of pharmacovigilance data. These studies have generated valuable data on pregabalin's prescribing patterns, adverse effects, and risk assessments. In Germany, the study conducted by Grosshans M [13] addressed the psychiatric effects and clinical toxicology of pregabalin, providing important insights into its applications in the field of forensic medicine.

Another significant focus in the literature is field-based research conducted in Middle Eastern and North African countries. Studies conducted in countries such as Saudi Arabia, Jordan, and Egypt have primarily focused on user experiences, the uncontrolled prescribing of the drug, and the level of pharmaceutical knowledge. In this context, qualitative studies conducted by Wazaify M and Van Hout MC within the Jordan-Ireland framework have highlighted the influence of cultural context on pregabalin misuse [5].

Türkiye's contribution to the literature has gained momentum in recent years. The publications originating from Türkiye primarily focus on two main themes: the non-prescription availability of pregabalin and the increasing patterns of misuse among young adults, as well as case-based analyses rooted in clinical psychiatry and forensic medicine [16] [17]. However, research activities in Türkiye have not yet positioned the country at the centre of the global collaboration network. This situation could be reversed through more active participation in international projects and diversification of research funding sources.

Highly cited studies are generally composed of systematic reviews, multicenter collaborations, or forensic toxicology analyses based on fatal case reports. The works of authors such as *Schifano F*, *Ojanperâ I*, and *Hâkkinen M* are not only academically influential but also serve as key references in shaping health policies. Notably, Schifano's 2014 publication has influenced the pharmacovigilance policies of the European Medicines Agency (EMA) and was taken into consideration during the reclassification process of pregabalin [1].

At the journal level, prominent publication outlets include the B*ritish Journal of Clinical Pharmacology, CNS Drugs, Addiction*, and F*orensic Science International*. These journals offer a balanced perspective on both the therapeutic effects and the misuse potential of the drug, thereby serving as a common platform for researchers from diverse disciplines [10] [12].

## Conclusion

This bibliometric analysis revealed a marked intensification of academic interest in pregabalin misuse and dependence, particularly over the past decade. While the majority of publications originate from countries with high research capacity, such as the US, the UK, and Germany, there has also been a noticeable increase in studies on pregabalin misuse in developing countries such as Türkiye. The most prolific authors and institutions tend to cluster around specific academic networks, indicating a centralisation of both scientific focus and research funding.

Pregabalin is frequently discussed in the scientific literature not only for its therapeutic potential, but also for its risk of misuse and its implications at the level of pharmacovigilance. Collaborations among authors and between countries reflect the multidisciplinary nature of the subject and facilitate the conduct of more comprehensive analyses.

Future research focusing on patterns of misuse among the youth population, prescribing policies, and the interactions of pregabalin with opioids will contribute to the development of more effective public health strategies. In the context of Türkiye, enhancing healthcare professionals' awareness and promoting multidisciplinary data sharing are expected to deepen scientific contributions in this field.

At this point, the role of biochemistry laboratories is of critical importance. Strengthening laboratory-based toxicological analyses for the detection, monitoring, and clinical interpretation of substances such as pregabalin is of vital importance for patient safety as well as for forensic and epidemiological surveillance. Therefore, biochemistry laboratories responsible for substance analysis have a critical role to play, and strengthening these units in terms of infrastructure, technical capacity, and personnel will enhance the healthcare system's resilience against such pharmacological risks.

## Dodatak

### Conflict of interest statement

All the authors declare that they have no conflict of interest in this work.
